# Morphometric analysis of the Filipino knee and its implication in total knee arthroplasty prosthesis design

**DOI:** 10.1186/s42836-022-00117-8

**Published:** 2022-04-05

**Authors:** Cleff Lucero Flores, Jose Antonio G. San Juan

**Affiliations:** Department of Orthopaedics, Chong Hua Hospital-Cebu City, Don Mariano Cui St., 6000 Cebu City, Philippines

**Keywords:** Filipino knee morphometry, Prosthesis design, Total knee arthroplasty

## Abstract

**Background:**

Prosthesis factors account for a quarter of the dissatisfaction rates among post-total knee replacement (TKR) patients. In the Philippines, the available prostheses have pre-determined sizes and dimensions that are based on Caucasian morphometric data. This can pose a problem, since according to previous studies Asian knees have smaller dimensions compared to Caucasians. Since there is a paucity of research looking into the fitness of these prostheses to the Filipino knee, this study was pursued.

**Methods:**

This study measured 675 knees of 675 adult Filipinos from January 2018 to December 2020. The morphometric measurements were performed on T1-weighted magnetic resonance images. The distal femoral morphometry included: the anteroposterior distance, lateral and medial anteroposterior distances, mediolateral distance, anterior and posterior mediolateral distances, and the femoral aspect ratio. The proximal tibial morphometry included: the anteroposterior distance, mediolateral distance, the medial and lateral anteroposterior distances, and the tibial aspect ratio. The patellar height, width, and thickness were also determined. Statistical analyses were done by using SPSS (version 26) and Microsoft Excel (version 2016).

**Results:**

The mean femoral medial and lateral anteroposterior distances were 57.6 mm and 57.1 mm, respectively. The femoral mediolateral distance was 69.3 mm, and the mean femoral aspect ratio was 1.21. The mean proximal tibial antero-posterior and mediolateral distances were 45.3 mm and 71.9 mm, respectively. The mean tibial aspect ratio was 1.66. Most TKR prostheses can be fitted to the Filipino knee but underhang on the mediolateral aspect is commonly observed in both the femoral and tibial components. The mean patellar height and width of Filipinos were 39.6 mm and 42.6 mm, respectively while the average thickness was 23.1 mm.

**Conclusion:**

Most prostheses available in the Philippine and Asian markets can be fitted into Filipino knees albeit the underhang observed in the mediolateral aspects of both femoral and tibial components. Potential patellar complications are unlikely given the adequate thickness. To avoid the potential mismatch, the best approach is to design a prosthesis aptly suited for the Filipino knees.

## Background

Total knee replacement (TKR) is an effective surgical procedure in addressing symptomatic end-stage osteoarthritis [1]. However, up to 25% of post-TKR patients are dissatisfied with their results. This dissatisfaction could be attributed to prosthesis factors [2]. Currently, most of the total knee prostheses have predetermined dimensions which are usually based on the Caucasian morphometric data. Many authors have highlighted the discrepancies between these prostheses and the Asian knee dimensions. They emphasized that Asian knees are smaller than their Caucasian counterparts, and “Asian” dimensions must be considered in prosthesis design [1, 3]. The diversity in social and cultural practices from squatting, kneeling, and ambulation complicates the demand that patients expect from the reconstructed knee. Thus, a more in-depth understanding of knee anatomy among ethnic groups must be pursued to address their unique expectations. In literature, the Asian population is well-represented by the Chinese, Japanese, and Koreans but the Southeast Asian population particularly the Filipinos have limited representation. Thus, this study was pursued to investigate the possible mismatch between the different prosthesis systems available in the Philippine and Asian markets and the Filipino knee dimensions.

## Materials and methods

**Fig. 1:**
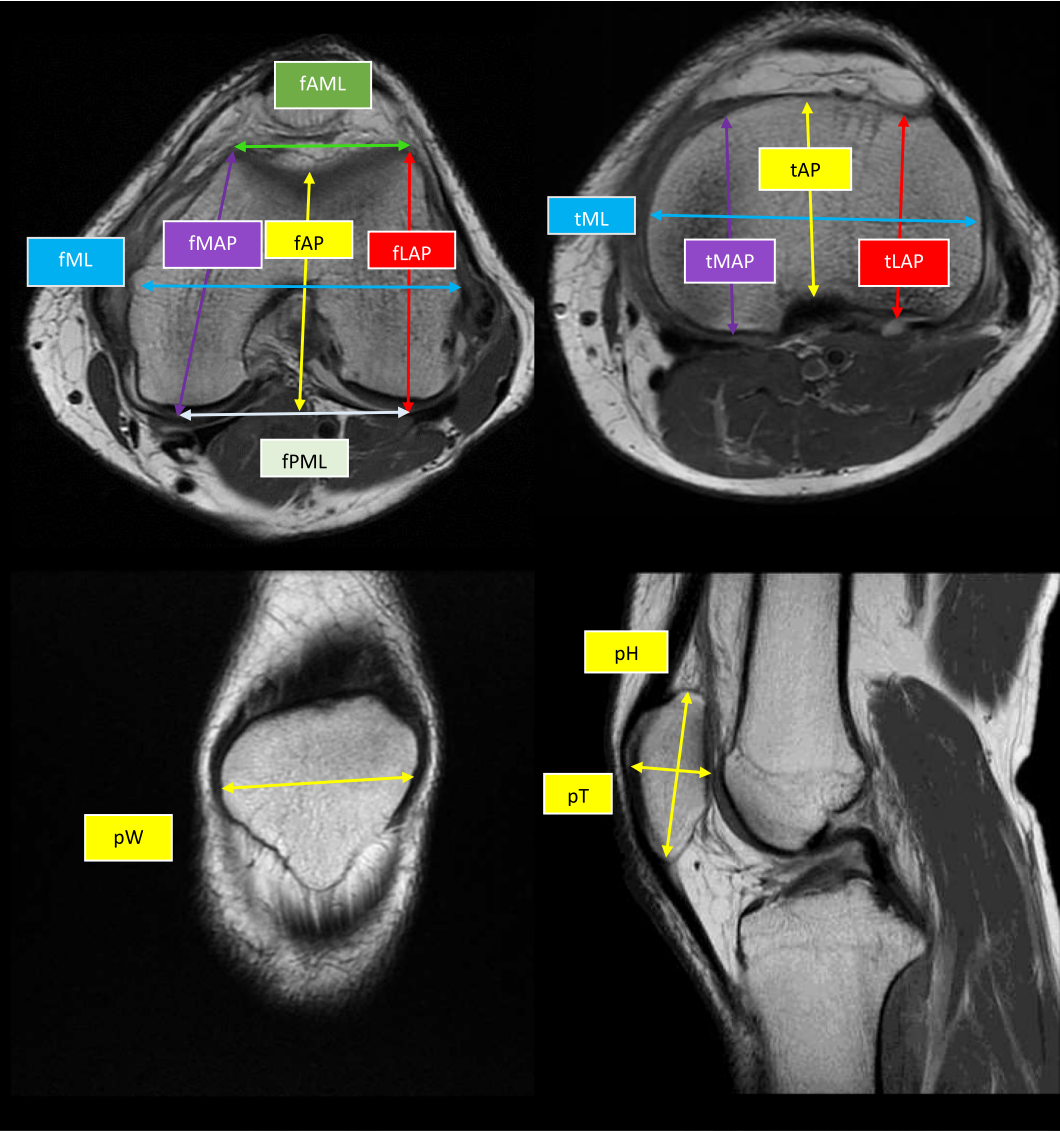
Morphometric measurements of the knee

From January 1, 2018 to December 31, 2020, 675 knees of 675 adult Filipinos were retrospectively reviewed. We examined the knees on T1-weighted magnetic resonance imaging (MRI) and measured the morphometric parameters of the distal femur, proximal tibia, and patella. Excluded were the knees with osseous pathologies, fractures and deformities. The knee MRIs were taken using the 1.5 T Siemens Aera MRI unit (Enlargen, Germany) at a thickness of 3 mm and an interval of 0.3 mm. Morphometric measurements were performed at the level of 9 to 10 mm proximal to the joint line and 9 to 10 mm distal to the joint line. The measurements were made by using the StarPACS, Infinitt software package (Seoul, South Korea) to the nearest 0.1 mm. The primary author (CF) and two CT-MRI fellows independently measured the parameters which are presented and defined in Fig. 1 and Table 1, respectively. The interclass correlation coefficient (ICC) was determined to evaluate the degree of correlation of the values recorded by the three raters.

**Table 1:** The morphometric parameters of distal femur, proximal tibia and the patella

Abbreviation	Measurement	Definition
fAP	Femoral antero-posterior distance [4, 5]	Distance from the deepest point of the trochlea to the fPML line that is tangential to the posterior femoral condyles
fML	Femoral medio-lateral distance [5–7]	Distance from the medial-most to the lateral-most aspects of the distal femur at the level of the intercondylar notch
fLAP	Femoral lateral antero-posterior distance^.^ [3–5]	Distance from the anterior-most to the posterior-most aspects of the lateral femoral condyle
fMAP	Femoral medial antero-posterior distance [1, 3–5]	Distance from the anterior-most to the posterior-most aspects of the medial femoral condyle
fAML	Femoral anterior medio-lateral distance [5]	Distance between 2 anterior-most points of the medial and lateral femoral condyles
fPML	Femoral posterior medio-lateral distance [5]	Distance between 2 posterior-most points of the medial and lateral femoral condyles
fAR	Femoral aspect ratio [6]	The quotient of fML and the average of the fMAP and fLAP
tML	Tibial medio-lateral distance [3–5]	The distance from the medial-most to the lateral-most aspects of the proximal tibia on cross-sectional view
tAP	Tibial antero-posterior distance [3–5]	The distance from the anterior-most to the posterior-most aspects of the tibial plateau passing through the midpoint of the intercondylar eminence
tLAP	Tibial lateral antero-posterior distance [1, 3, 5]	The distance from the anterior-most to the posterior-most aspects of the lateral tibial condyle
tMAP	Tibial medial antero-posterior distance [1, 3, 5]	The distance from the anterior-most to the posterior-most aspects of the medial tibial condyle
tAR	Tibial aspect ratio [6]	The quotient of tML and the average of the tMAP and tLAP
pH	Patellar height	The distance from the superior-most to the inferior-most aspects of the patella on sagittal view with the profile of the patella at its maximum
pW	Patellar width	The distance from the medial-most to the lateral-most aspects of the patella on coronal view with the maximum outline of the patella
pT	Patellar thickness	The distance from the anterior-most to the posterior-most aspects of the patella on sagittal view with the profile of the patella at its maximum

### Comparison between prostheses in the Philippine and Asian markets

The currently available prostheses in the Philippine and Asian markets were reviewed. The dimensions of the prostheses and knees were plotted. The best fit line was used to observe the fitness of each prosthesis *vis*-*à*-*vis* the dimensions of the Filipino knee. The following implants were used for comparison: (1) Advance MPK (Microport Orthopedics, TN, USA); (2) Axis Knee (DOST-PCHRD, Philippines); (3) Duracon (Stryker Corp., MI, USA); (4) Gemini (Waldemar Link GmbH & Co., Germany); (5) Genesis II and Legion (Smith and Nephew, UK); (6) PFC Sigma (DePuy-Synthes, IN, USA); (7) Scorpio (Stryker Corp., MI, USA); and (8) U2 Knee (United Orthopaedics Corp., Taiwan). The aspect ratios of these prostheses were respectively based on the specifications provided by their corresponding companies.

### Data collection and statistical analysis

The interclass correlation coefficient (ICC) was computed to determine the extent of agreement between the 3 MRI raters. The average value of the 3 raters was recorded in the final data. SPSS (version 26) and Excel (Microsoft 2016) were utilized for statistical analyses. The independent student *t*-test was used to determine the significance of the differences in the morphometric measurements between the sexes. Pearson correlation test was employed to measure the correlation of each morphometric parameter with each other. The statistical significance was set at *P* < 0.05.

## Results

The demographic data are presented in Table 2. The difference in age between males and females was found to be significant (*P* < *0.*01).

**Table 2:** Demographic data

Parameter	Value (years)			(*P*-Value)
	**Combined**	**Male** *n* = 348	**Female** *n* = 327	
Age	43.1 ± 15.15	38.2 ± 14.1	48.3 ± 14.5	9.14 ^a^ (< 0.001)

Values are presented in mean ± SD, ^a^ Significance was set at 0.05 as tested by *t*-test for two independent samples.

### Morphometry of distal femur

Table 3 shows the morphometric measurements for the distal femur. The average femoral anteroposterior distance (fAP) for both sexes was 48.9 mm. The difference of fAP distances between males and females was statistically significant (*P* < *0.*001). The mean femoral medial and lateral anteroposterior distances (fMAP and fLAP) for both sexes were 57.6 mm and 57.1 mm, respectively. Males tended to have greater values than females (*P* < 0.001).

**Table 3:** Morphometric measurements of the distal femur

Parameters	Value (in millimeters)			*P* value
	**Combined sex** (*n* = 675)	**Male** (*n* = 348)	**Female** (*n* = 327)	
fAP	48.9 ± 3.4	50.3 ± 3.1	47.4 ± 3.0	< 0.001
fMAP	57.6 ± 4.5	60.0 ± 4.1	54.9 ± 3.2	< 0.001
fLAP	57.1 ± 4.6	59.7 ± 4.1	54.4 ± 3.4	< 0.001
fML	69.3 ± 6.7	74.3 ± 4.7	64.1 ± 3.9	< 0.001
fAML	37.3 ± 4.3	39.8 ± 3.7	34.6 ± 3.2	< 0.001
fPML	49.6 ± 5.4	52.9 ± 4.6	46.2 ± 3.7	< 0.001
fAR	1.21 ± 0.07	1.24 ± 0.07	1.17 ± 0.06	< 0.001

Values are presented as mean ± SD, *Significance was set at 0.05 as tested by student *t*-test for two independent samples.

The average femoral mediolateral distance (fML) was 69.3 mm for both sexes, and males had greater values than females (*P* < 0.001). There was also a statistically significant difference in the femoral aspect ratios (fAR) between two sexes (*P* < 0.001).

### Morphometry of proximal tibia

Table 4 presents the values of the proximal tibial parameters. The mean AP distance of tibia was 45.3 mm while the mean mediolateral distance was 71.9 mm. The mean medial and lateral AP distances were 43.9 mm and 42.9 mm, respectively. Males registered statistically greater values than females in all parameters except in the tibial aspect ratio (*P* = 0.088).

**Table 4:** Morphometric measurements of the proximal tibia

Parameters	Value (in millimeters)			(*P*-Value)
	**Combined**	**Male** *n* = 348	**Female** *n* = 327	
tAP	45.3 ± 4.3	47.9 ± 3.5	42.5 ± 3.0	< 0.001
tML	71.9 ± 10.8	76.7 ± 12.9	66.8 ± 3.8	< 0.001
tMAP	43.9 ± 4.1	46.4 ± 3.5	41.4 ± 2.9	< 0.001
tLAP	42.9 ± 4.3	45.6 ± 3.5	40.0 ± 2.88	< 0.001
tAR	1.66 ± 0.19	1.67 ± 0.25	1.65 ± 0.09	= 0.088

Values are presented as mean ± SD, * Significance was set at 0.05 as tested by student *t*-test for two independent samples.

### Morphometry of patella

The average patellar height was 39.6 mm while the mean patellar width was 42.6 mm (Table 5). The average patellar thickness was 23.1  mm. Filipino males had longer, wider and thicker patellae than females (*P* < 0.001).

**Table 5:** Morphometric measurements of the patella

Parameters	Value (in millimeters)			*P*-Value
	**Combined**	**Male** *n* = 348	**Female** *n* = 327	
pH	39.6 ± 4.7	42.1 ± 3.8	36.9 ± 3.9	< 0.001
pW	42.6 ± 4.2	45.0 ± 3.5	40.0 ± 3.3	< 0.001
pT	23.1 ± 4.4	24.2 ± 2.1	21.9 ± 5.7	< 0.001

Values are presented as mean ± SD, *Significance was set at 0.05 as tested by student *t*-test for two independent samples.

### Interclass correlation coefficient and correlation of morphometric parameters

The interclass correlation coefficient between MRI raters revealed good reliability (Table 12 in Appendix). In Table 13 (Appendix) the correlation between each parameter is presented. The femoral ML distance was found to have strong positive association to both the femoral medial AP distance and the femoral lateral AP distance. The tibial AP distance was found strongly correlated to the tibial lateral AP distance but only moderately correlated to the tibial ML distance and the tibial medial AP distance. The tibial aspect ratio (tAR) was moderately correlated to the tibial ML distance but was negligibly correlated with the rest of the tibial parameters.

### TKR prosthesis dimensions

**Fig. 2:**
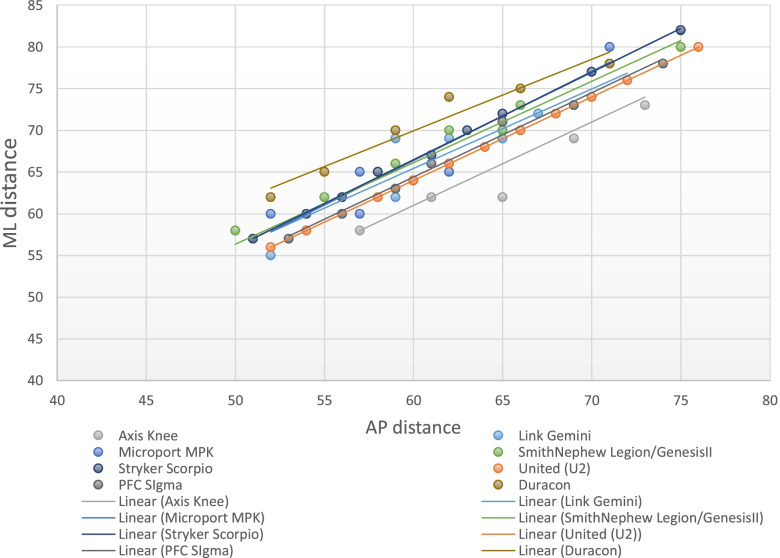
Anteroposterior and mediolateral dimensions of TKR femoral prostheses in the Philippine and Asian markets

Table 6 and Fig. 2 present the AP and ML dimensions of the available TKR femoral prostheses in the Philippine and Asian markets. We observed a positive slope in the best-fit lines of all the prostheses approaching 1.0 (Fig. 2). This means that the rates of change in both AP and ML dimensions are equal. Thus, the shape has not changed with the increase of prosthesis size.

**Table 6:** Anteroposterior and mediolateral dimensions of TKR-femoral prostheses in the Philippine and Asian markets (in millimeters)

	Axis- PCHRD		Duracon Stryker		Gemini- Link		Genesis II Smith&Nephew		Advance MPK-Microport		PFC Sigma DePuy		Scorpio Stryker		U2-United	
Size^a^	AP	ML	AP	ML	AP	ML	AP	ML	AP	ML	AP	ML	AP	ML	AP	ML
A	57	58	52	62	52	55	50	58	52	60	53	57	51	57	52	56
B	61	62	55	65	55	62	54	58	57	60	56	60	54	60	54	58
C	65	62	59	70	59	62	55	62	57	65	59	63	56	62	56	60
D	65	69	62	74	59	69	58	62	62	65	61	66	58	65	58	62
E	69	69	66	75	62	69	59	66	62	70	65	71	61	67	60	64
F	69	73	71	78	65	69	61	66	66	70	69	73	63	70	62	66
G	73	73			67	72	62	70	66	75	74	78	65	72	64	68
H					72	76	65	70	71	80			70	77	66	70
I							66	73					75	82	68	72
J							70	77							70	74
K							75	80							72	76
L															74	78
M															76	80

^a^No uniform sizing categories exist among companies.

**Fig. 3:**
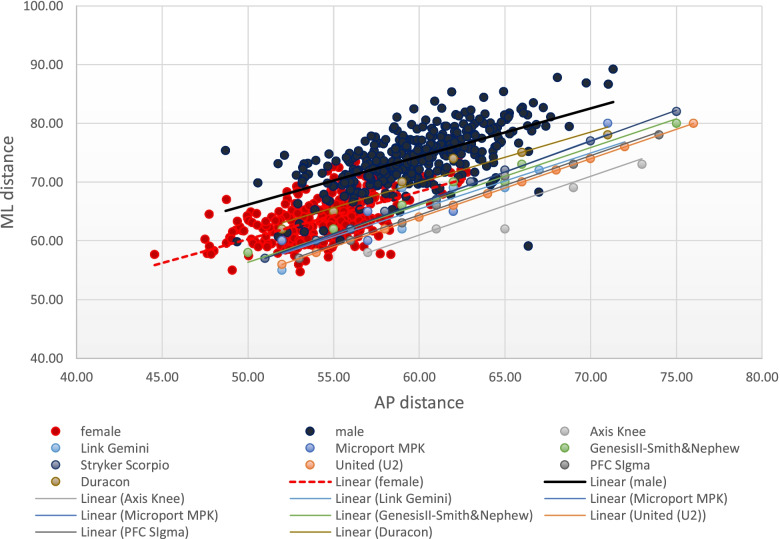
The anteroposterior and mediolateral dimensions of the Filipino distal femur and the TKR femoral prostheses

Fig. 3 illustrates the trend of the AP and ML dimensions of the distal femur as plotted with the

AP and ML dimensions of the different prostheses. There is a mismatch between the distal femur morphology and the dimensions of the eight implant systems. Most Filipino knees had an AP distance between 50 to 70 mm and an ML distance commonly ranging from 55 to 85 mm. For a given AP dimension of most implants, the ML aspect tends to be smaller relative to the Filipino knee. Thus, there is a propensity for underhang.

**Fig. 4:**
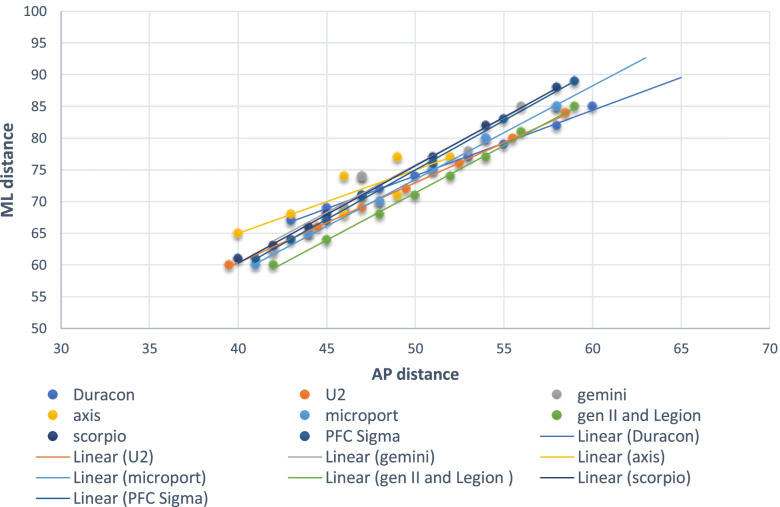
Anteroposterior and mediolateral dimensions of TKR tibial prostheses in the Philippine and Asian markets

**Fig. 5:**
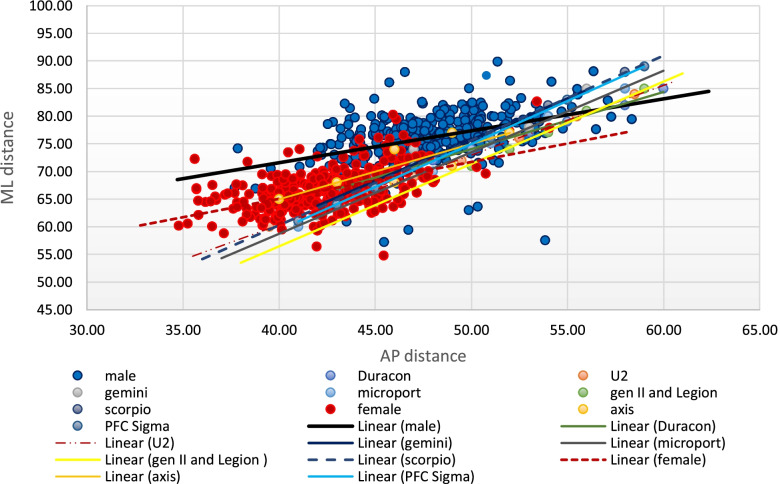
The AP and ML dimensions of the Filipino proximal tibia and their correlation with TKR tibial prostheses

Table 7 and Fig. 4 show the anteroposterior and mediolateral dimensions of the available TKR tibial implants in the Philippine and Asian markets. Figure 5 illustrates the trend of the proximal tibial dimensions as plotted with the AP and ML dimensions of the different tibial prostheses. A mismatch can be observed between the proximal tibial dimensions and the dimensions of the eight tibial prostheses. Most of the implants shared the same slope approaching the value of 1.5. Most Filipino proximal tibias had an AP distance between 35 and 55 mm and an ML distance ranging from 60 to 85 mm. Just like in the distal femur, there is a propensity for underhang in the mediolateral aspect when a properly-sized tibial component was fitted to the native proximal tibia.

**Table 7:** Anteroposterior and mediolateral dimensions of the available TKR-tibial prostheses in the Philippine and Asian markets (in millimeters)

	Axis- PCHRD		Duracon Stryker		Gemini- Link		Genesis II Smith&Nephew		MPK-Microport		PFC Sigma DePuy		Scorpio Stryker		U2-United	
Size^a^	AP	ML	AP	ML	AP	ML	AP	ML	AP	ML	AP	ML	AP	ML	AP	ML
A	57	58	52	62	52	55	50	58	52	60	53	57	51	57	52	56
B	61	62	55	65	55	62	54	58	57	60	56	60	54	60	54	58
C	65	62	59	70	59	62	55	62	57	65	59	63	56	62	56	60
D	65	69	62	74	59	69	58	62	62	65	61	66	58	65	58	62
E	69	69	66	75	62	69	59	66	62	70	65	71	61	67	60	64
F	69	73	71	78	65	69	61	66	66	70	69	73	63	70	62	66
G	73	73			67	72	62	70	66	75	74	78	65	72	64	68
H					72	76	65	70	71	80			70	77	66	70
I							66	73					75	82	68	72
J							70	77							70	74
K							75	80							72	76
L															74	78
M															76	80

^a^No uniform sizing category exist among companies.

## Discussion

### Morphometry of distal femur

In the current study, the average femoral medial and lateral AP distances, which were 57.6 mm and 57.1 mm respectively, are comparable to those observed in Indians, Koreans and Chinese knees [6, 8, 9]. These values, however, are lesser than those recorded by Kim *et al*. [1] for the East Asian populations (represented by the Chinese, Japanese, Koreans, Malaysians, and Thais). The Filipino average fMAP and fLAP are also greater than the Indonesian average but lesser compared to the Malaysian average values [10, 11]. Previous studies reported that Caucasian knees had greater distal femur dimension as compared to Asian ethnicities (Table 8) [1, 5, 10]. The current study registered an average value of 69.3 mm for the mediolateral distance of the distal femur which is comparable to that reported by Mohan *et al.* [6] for the Indian knees and Chaichankul *et al.* [12] for the Thai average. In comparison, Indonesian and Malaysian knees had lesser fML values while Koreans and other East Asian populations (Chinese, Japanese, Koreans) had higher values [1, 8, 10, 11]. The Caucasian population consistently had greater fML values in multi-ethnicity comparative studies [1, 5, 9]. Only a number of studies reported on the anterior and posterior mediolateral distances (fAML and fPML) of the distal femur. These parameters are deemed important in determining the total shape of the femur [13]. A narrow fAML and a wider fPML describe a trapezoidal distal femur on cross sectional view. Fan *et al.* [5] reported a very comparable fAML and fPML averages for the Southern Chinese population while Mahfouz* et al.* [14] reported a lesser fAML average and greater fPML average for Caucasians and African Americans.

**Table 8:** Femoral morphometric parameters of different ethnicities (in millimeters)

	**fMAP**	**fLAP**	**fML**	**fAML**	**fPML**	**fAR**
Cheng *et al.* (Chinese) [3]	F: 49.8 ± 3.2 M: 52.6 ± 2.4 Combined: 51.3 ± 3.3	F: 49.3 ± 4.1 M: 51.8 ± 3.7 Combined: 50.7 ± 4.0	F: 66.8 ± 3.1 M: 74.4.6 ± 29 Combined: 71.0 ± 3.0	Not reported	Not reported	F: 1.10 ± 3.6 M: 1.12 ± 3 Combined 1.11 ± 2.7
^a, b^Chaichankul *et* *al.* (Thai) [12]	F: 43.32 ± 3.7 M: 48.55 ± 3.7 Combined:45.4		F: 59.91 ± 3.75 M: 70.1 ± 3.87 Combined: 65.0	Not reported	Not reported	F:1.39 ± 1.2 M: 1.45 ± 1.1 Combined: 1.41 ± 1.2
Mahfouz *et al.* (African American) [14]	F: 63.9 ± 6.5 M: 66.9 ± 3.5	F: 64.1 ± 4.9 M: 71.1 ± 3.5	F: 76.8 ± 4.9 M: 84.9 ± 4.7	F: 31.16 ± 6 M: 38.1 ± 3.6	F: 46.7 ± 4 M: 52.1 ± 5.1	F: 1.38 ± 0.34 M: 1.39 ± 0.07
Mahfouz* et al.* (Caucasian) [14]	F: 59.4 ± 3.3 M: 65.7 ± 3.7	F: 61.4 ± 3.2 M: 67.8 ± 4.1	F: 75.8 ± 3.3 M: 85.9 ± 4.7	F: 29.9 ± 2.9 M: 34.4 ± 3.5	F: 46.9 ± 2.9 M: 53.5 ± 4.2	F: 1.36 ± 0.06 M: 1.41 ± 0.06
Mahfouz *et al.* (Asian) [14]	F: 56.4 ± 3 M: 62.6 ± 3.8	F: 57.8 ± 3.2 M: 64.8 ± 4.4	F: 74.8 ± 3.3 M: 85.4 ± 4.3	F: 31.8 ± 2.3 M: 37.0 ± 2.9	F: 44.8 ± 3.3 M: 50.9 ± 5	F: 1.5 ± 0.1 M: 1.56 ± 3.8
Lim *et al.* (Korean) [8]	F:56.8 ± 3.31 M: 62.7 ± 4.10	F: 58.4 ± 3.10 M:59.0 ± 4.01	F: 76.7 ± 3.71 M:81.5 ± 5.70	Not reported	Not reported	F: (fML/fMAP): 1.31 (fML/fLAP): 1.35 M: (fML/fMAP): 1.30 (fML/fLAP): 1.38
Fan *et al.* (Southern Chinese) [5]	F: 59.6 ± 3.6 M: 64.9 ± 3.5	F:58.3 ± 3.9 M: 64.0 ± 3.8	F: 71.1 ± 3.6 M: 80.6 ± 3.5	F: 33.8 ± 2.3 M: 38.6 ± 2.6	F: 46.3 ± 3.0 M: 51.8 ± 3.5	F:1.23 ± 0.07 M: 1.27 ± 0.07
^b^Mohan *et al.* (Indian) [6]	F: 52.8 ± 3.13 M: 57.52 ± 3.12 Combined: 55.73 ± 3.87		F: 64.75 ± 3.37 M: 73.74 ± 4.07 Combined: 70.32 ± 5.8	Not reported	Not reported	F:1.23 ± 0.07 M: 1.28 ± 0.07
^b^Mohan *et al.* (Chinese) [6]	F: 52.80 ± 2.6 M: 56.50 ± 2.5		F: 64.4 ± 2.6 M: 72.7 ± 3.8	Not reported	Not reported	F:1.22 ± 0.05 M: 1.29 ± 0.05
^b^Mohan *et al.* (Hispanic) [6]	F: 45.6 ± 3.2 M: 49.9 ± 3.8		F: 66.3 ± 3.0 M: 77.2 ± 4.10	Not reported	Not reported	F:1.46 ± 0.09 M: 1.55 ± 0.11
^b^Mohan *et al.* (Caucasian) [6]	F: 55.4 ± 2.8 M: 59.6 ± 3.2		F: 65.4 ± 1.4 M: 74.6 ± 3.9	Not reported	Not reported	F:1.25 ± 0.05 M: 1.18 ± 0.05
Current Study (Filipino)	F: 54.9 M: 60.0 Combined: 57.6 ± 4.5	F: 54.4 M: 59.7 Combined: 57.1 ± 4.6	F: 64.1 M:74.3 Combined: 69.3 ± 6.7	F: 34.6 M:39.8 Combined: 37.3 ± 4.3	F:46.2 M: 52.9 Combined: 49.6 ± 5.4	F: 1.17 ± 0.6 M: 1.24 ± 0.7 Combined: 1.21 ± 0.07

^a^resected bone

^b^the mean of fMAP and fLAP

#### Femur surface ratio

The femoral surface aspect ratio (fAR) provides a rough estimate of the shape of the distal femur and is considered an important determinant of the suitability of the prosthesis to the native knee. A higher aspect ratio corresponds to a wider ML dimension relative to the AP dimension. This can be observed in smaller knees. Conversely, a lower aspect ratio can be observed in larger knees as shown by Hitt *et al*. [15] The average fAR in this series was 1.21 ± 0.1, which is lower than that found among Southern Chinese, Koreans and Indians [5, 6,14].  In a multi-ethnicity study, Mahfouz *et* *al.* [14] found that Asians had a higher fAR compared to the Caucasians and Africa-Americans. These observations highlighted the influence of ethnicity in the total shape of the distal femur and must be a factor to consider when designing a prosthesis. Kim *et al.*^.^ [1] also made the same observation that East Asian knees (Chinese, Japanese, Koreans, Thais and Malaysians) had significantly smaller AP dimension than White and Black ethnicities. The femoral morphometry not only varies among ethnicities but between sexes of every ethnicity. Fan *et al.* [5] reported a lower aspect ratio in narrower distal femur, which is typical of the female knees. In the current report, Filipino males consistently had higher femoral morphometric values compared to females.

**Fig. 6:**
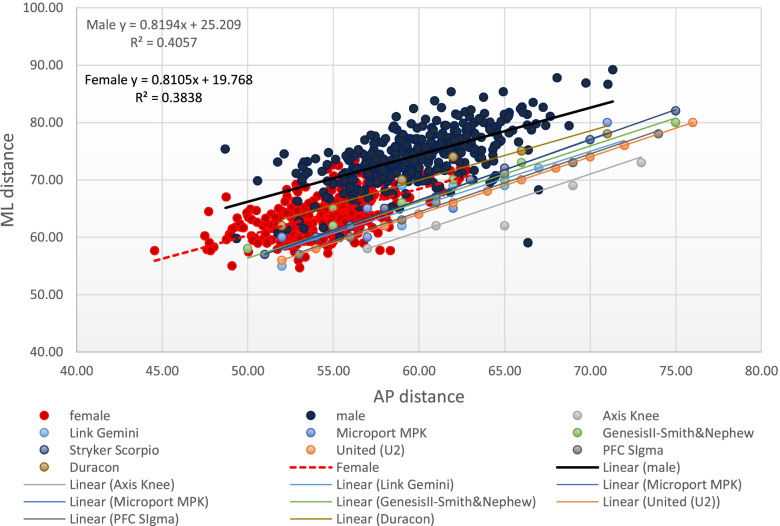
The difference in slope between the native knees and the TKR-femoral prostheses

**Fig. 7:**
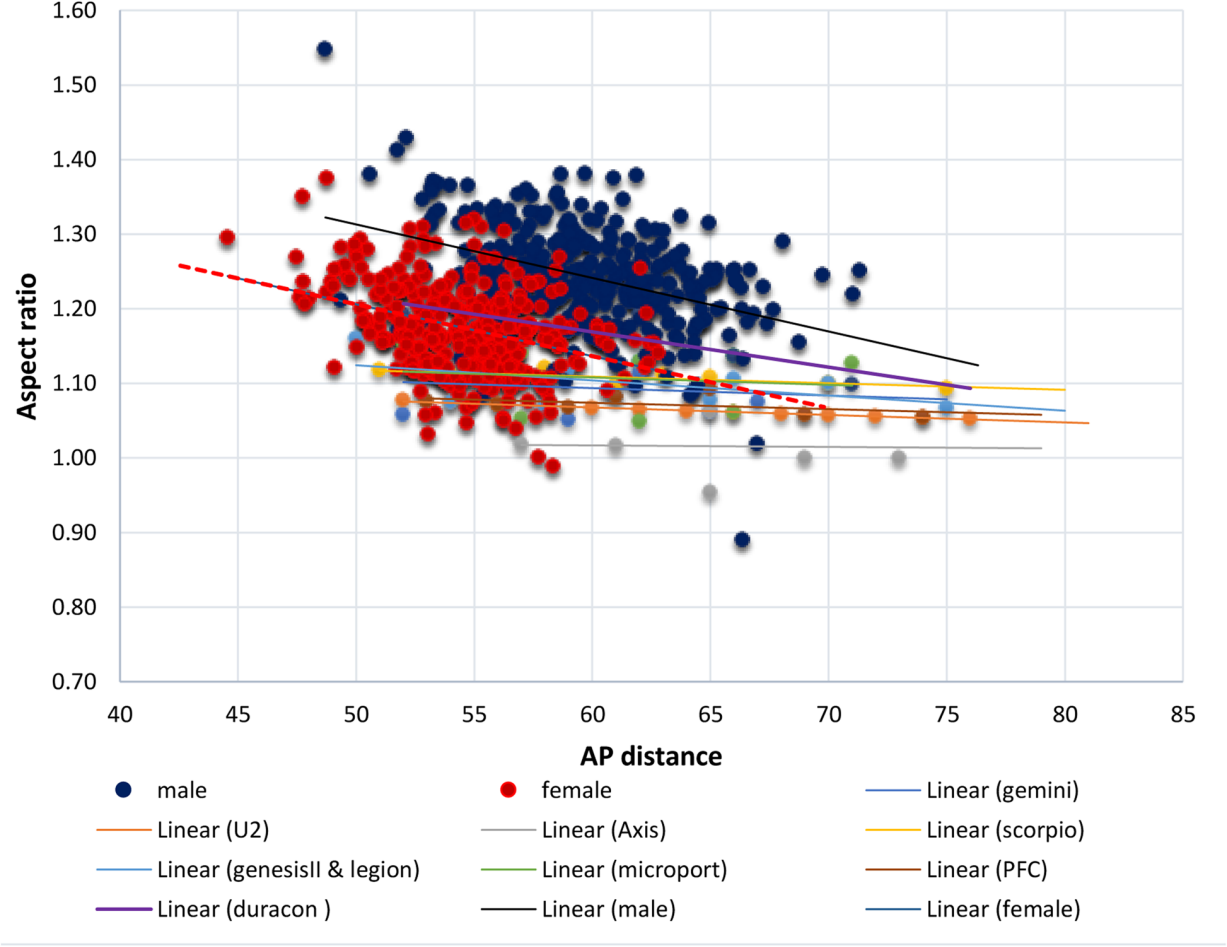
The aspect ratio to AP distance of the femoral prostheses

Only one of the eight prostheses is manufactured locally in the Philippines (Axis Knee® System) while the rest are sourced internationally. When the AP and ML dimensions of these prostheses are plotted in a graph (Figs. 3 and 5), the best fit lines of each device showed a linear relationship between the AP dimension and the ML dimension, which were steeper as compared to the slope observed in the native knees. This creates a propensity for a mismatch since the increase in mediolateral aspect is not complemented by the same increase in the anteroposterior dimension which is demonstrated in Figs. 6 and 7. The shape of most prostheses remained constant or changed very minimally as their sizes increased. The implant Duracon® had a decreasing trend line comparable to the native knee (Fig. 7). The same observation was also reported by Hitt *et al.* [15] and Cheng *et al.* [3] in their respective series. While all sizes of the Filipino knees can be accommodated by the prostheses available in the Asian and Philippine markets, most knees fall within the “small to medium” size range based on the AP diameter. Most male AP values are within the range of 55 to 65 mm (77.6%), while most of the female knees are between 50 to 60 mm (94.2%). Despite the smaller knees of the Filipino, underhang on the mediolateral aspect is to be expected when using an appropriately-sized femoral prosthesis in the AP dimension (Fig. 6). The Axis knee system® will have the most underhang with a total of 13 mm or 6.5 mm on either side while Duracon® will have the least underhang with a 1 mm total underhang or a 0.5 mm on either side of the distal femur. Other implants (Gemini-Link, Genesis II-Smith&Nephew, Advance MPK-Microport) will have a total underhang ranging between ≤ 3 mm to ≤ 6 mm.

#### Proximal tibial morphometry

Table 9 demonstrates the average tibial AP distance of the Filipinos which is comparable to that reported in Korean, Indian and Thai populations [6, 12, 16]. Filipino males had higher values than females (*P* < 0.05). The same gender difference was observed by other authors [3, 5, 6, 9, 13, 14]. The mean tibial ML distance was 71.9 mm for both sexes. The value observed in males was significantly higher than that in females (*P* < 0.001). Comparable values were reported by earlier studies conducted in Korean, Thai and Indian knees [6, 10, 12].

**Table 9:** Tibial morphometric parameters of different ethnicities (in millimeters)

	tAP	tMAP	tLAP	tML	tAR
Kwak *et al.* (Korean) [13]	F: 43.2 ± 2.3 M: 48.2 ± 3.3 Combined: 45.7 ± 3.8	F: 43.5 ± 3.7 M: 48.5 ± 3.7 Combined: 45.9 ± 4.2	F: 39.8 ± 2.5 M: 43.5 ± 2.9 Combined: 45.9 ± 4.2	F: 67.6 ± 3.1 M: 76.1 ± 4.0 Combined: 71.9 ± 5.6	F: 1.6 M: 1.6 Combined: 1.8
Cheng *et al.* (Chinese) [3]	F: 45.7 ± 1.9 M: 51.3 ± 2.0 Combined: 48.8 ± 3.4	F: 47.5 ± 2.4 M: 53.3 ± 2.5 Combined: 50.7 ± 2.4	F: 42.4 ± 2.3 M: 47.7 ± 2.7 Combined: 45.3 ± 2.5	F: 68.8 ± 4.6 M: 76.4 ± 2.8 Combined: 73.0 ± 4.6	F: 1.52 ± 0.1 M: 1.49 ± 0.1 Combined: 1.49 ± .05
Chaichankul *et al.* (Thai) [12]	F: 43.23 ± 2.6 M: 50.15 ± 3.1 Combined: 46.04 ± 4.4	F: 59.6 ± 3.6 M: 64.9 ± 3.5	F:58.3 ± 3.9 M: 64.0 ± 3.8	F: 64.95 ± 2.6 M: 74.44 ± 3.4 Combined: 68.8 ± 5.8	F:1.5 M: 1.5 Combined: 1.5
Mahfouz *et al*. (African-American) [14]	F: 52.5 ± 3.5 M: 57.3 ± 3.7	F: 30.4 ± 6.3 M:21.0 ± 4.3	F: 23.0 ± 1.7 M: 23.0 ± 2.4	F: 66.2 ± 3.8 M: 79.3 ± 3.8	F: 1.26 ± 0.1 M: 1.39 ± 0.1
Mahfouz *et al*. (Caucasian) [14]	F: 50.0 ± 3.8 M: 56.8 ± 3.5	F: 19.4 ± 3.4 M: 22.6 ± 2.7	F: 19.3 ± 1.9 M: 22.0 ± 1.7	F: 68.6 ± 4.8 M: 79.24 ± 4.6	F: 1.37 ± 0.1 M: 1.40 ± 0.1
Mahfouz *et al*. (East Asian) [14]	F: 48.1 ± 3.3 M: 51.3 ± 3.8	F: 20.3 ± 0.9 M: 22.6 ± 1.7	F: 16.0 ± 1.5 M: 18.5 ± 1.9	F: 68.0 ± 3.0 M: 68.3 ± 6.8	F: 1.42 ± 0.1 M: 1.33 ± 0.1
Lim *et al.* (Korean) [8]	Not reported	F: 47.7 ± 3.0 M: 59.5 ± 5.0	F: 45.7 ± 3.3 M:52.7 ± 5.0	F: 70.0 ± 3.4 M:80.6 ± 6.3	F: (tML/tMAP)1.47 ± 0.04 (tML/tLAP)1.53 ± 0.04 M: (tML/tMAP)1.35 ± 0.05 (tML/tLAP) 1.53 ± 0.04
Fan *et al.* (Southern Chinese) [5]	Not reported	F: 59.6 ± 3.6 M: 64.9 ± 3.5	F:58.3 ± 3.9 M: 64.0 ± 3.8	F: 71.1 ± 3.6 M: 80.6 ± 3.5	F:1.23 ± 0.1 M: 1.27 ± 0.1
Mohan *et al.* (Indian) [6]	F:43.29 ± 2.7 M: 49.12 ± 3.8 Combined 46.9 ± 4.5	Not reported	Not reported	F: 65.52 ± 3.2 M: 75.66 ± 4.3 Combined: 71.8 ± 6.3	F:1.52 ± 0.1 M: 1.55 ± 0.1 Combined: 1.53 ± 0.1
Current Study (Filipino)	F: 42.5 ± 3.0 M: 47.9 ± 3.3 Combined: 45.3 ± 4.3	F: 41.4 ± 2.9 M: 46.4 ± 3.5 Combined: 43.9 ± 4.1	F: 40.0 ± 2.9 M: 45.6 ± 3.5 Combined: 42.9 ± 4.2	F: 66.8 ± 3.8 M: 76.05 ± 4.7 Combined: 71.88 ± 6.2	F: 1.65 ± 0.1 M: 1.67 ± 0.2 Combined: 1.66 ± 0.2

The Chinese tibial ML distance was found to be virtually identical to that of Filipinos but the Southern Chinese population have a significantly higher value [3, 5]. Generally, the tibial plateau is not symmetrical [5]. The tibial medial AP distance is usually longer than the lateral AP distance (Table 4). Hitt *et al.* [15] observed that the difference between tibial condyles was 5.2 mm in males and 4.3 mm in females. In Korean knees, the difference was 3.9 mm in males and 3.7 mm in females [16]. In the current series, the tMAP-tLAP difference was very marginal, being at 1.4 mm for females and 0.67 mm for males. There has been no study that investigated the effect of this asymmetry but some authors maintained that it may affect the total function of the reconstructed knee. Despite this, the minimal difference observed in the Filipino knees may not affect the ultimate functional outcome significantly.

The tibial aspect ratio just like in the distal femur is indicative of the shape of the proximal tibia on cross-sectional view. A larger value signifies a wide ML dimension relative to the AP distance. In the current study, the average tibial aspect ratio was 1.66, with males having higher value than females (*P* = 0.088). The AP and ML dimensions of the different tibial prostheses as well as the dimensions of the native proximal tibias are plotted in Fig. 5. Most Filipino female knees (74.6%) had a tAP distance ranging from 40 to 50 mm while Filipino males had a tAP distance ranging between 42 to 54 mm in 96.7% of the cases. Despite the observed small size, undercoverage can be expected as most implants have ML dimensions that are insufficient to fully cover the resected tibial plateau. For most implant systems with the exception of Duracon® and Axis knee System® (Table 7), undercoverage on the ML aspect is expected. This can range from 2.78 mm to 6.78 mm.

#### Patellar morphology

The patellar dimensions of Filipinos are presented in Table 10. The differences in patellar height and width between males and females were statistically significant (*P* < 0.001). These findings were also reported by Dorado-Fernandez *et* *al.* [17] who described a difference of 5.6 mm for the patellar height and a difference of 5.59 mm for patellar width between sexes. Filipinos have comparable patellar width and thickness with the Caucasians and Koreans while Indians had lower values [18–20]. Mohamed *et al.* [18] reported that the patellar thickness of Western ethnicities was 22.4 mm while South Chinese had an average thickness of 22.7 mm. These results are practically identical to our findings in Filipinos. Patella-related morbidities after TKR pose a major concern and the detailed morphometric knowledge can help in the development of an appropriately designed patellar component [19]. It is a common practice not to resurface patellae that are less than 20 mm in thickness to minimize the fracture risk. Also, failure to restore the native thickness of the patella during resurfacing may cause extension lag or patello-femoral joint morbidities. Kim *et al.* [19] stated that there are no adverse clinical or radiographic effects as long as the residual thickness of the bone is maintained between 10–15 mm or when the postoperative thickness is kept within 3 mm from its original thickness. For the majority (89%) of knees in this series, the patellar thickness is at least 20 mm. Most implant systems in Table 11 have a minimum patellar prosthesis thickness of 7 or 8 mm. Therefore, maintaining a bone thickness of at least 12 mm is not an issue for most Filipino knee.

**Table 10:** Patellar dimensions of different ethnicities (in millimeters)

	Patellar height	Patellar width	Patellar thickness
Kim *et al.* (Koreans) [19]	Female: 33.1 Male: 36.2 Combined: 34.65	Female: 41 Male: 45.6 Combined:43.3	Female:21.2 Male: 23.1 Combined: 22.2
Iranpour *et al.* (Caucasians) [20]	Combined: 34.4 ± 3.8	Combined:44.8 ± 4.8	Combined: 22.4 ± 2.3
Mohamed *et al.* (Indians) [18]	Female: 33.1 Male: 36.2 Combined: 33.1	Female: 36.1 Male: 42.2 Combined: 39.1	Female: 16.2 Male: 20.3 Combined: 18.3
Current study (Filipinos)	Female: 36.9 Male: 42.1 Combined: 39.6	Female: 40.0 Male: 45.0 Combined :42.6	Female: 21.9 Male: 24.3 Combined: 23.1

**Table 11:** Patellar diameter (D) and thickness (T) of different prosthesis systems (in millimeters)

	Gemini- Link		Genesis II Smith&Nephew		MPK-Microport		PFC Sigma DePuy		Scorpio Stryker		U2-United	
Size^a^	D	T	D	T	D	T	D	T	D	T	D	T
A	25	7	26	7.5/9	26	8	32	8	30	8	26	7
B	28	8	29	7.5/9	29	8	35	8.5	32	8	29	8
C	31	9	32	7.5/9	32	8	38	9	34	8	32	8.5
D	34	10	35	7.5/9	35	8	41	11.5	34	10	35	9
E					38	10			36	10	38	9.5
F					41	11			38	10	41	10
G											44	10.5

^a^No uniform sizing categories exist among companies.

### Influence of gender and ethnicity on knee morphometry

Filipino female knees are smaller compared to their male counterparts [1, 9]. Some authors disapprove of using “off-the-shelf” prostheses because unisex devices can potentially have suboptimal results. Efforts have been made to address the unique morphometries of both sexes. Despite the emergence of gender-specific TKR prosthesis, few clinical studies proved a relevant clinical advantage of these devices over the “unisex” variants aside from accomplishing the goal of proper bone coverage and reducing overhangs. East Asian knee morphometry is well-represented by the Chinese, Japanese and Koreans but only few studies have looked into the Southeast Asian populations. Hussain *et al.* [21] stated that the Malay populations, which include the Malaysian, Indonesian, and Singaporean are less represented in large studies. The authors attribute this to the lack of a robust number of cases and the generalization that Southeast Asians are of the same morphometries as the East Asians. East Asians and Southeast Asians share almost the same physical stature yet their cultural practices involving different postures of the knee may vary. Thus, the demand that they pose on the reconstructed knee may also vary. Efforts therefore must be made to develop a more aptly suited prosthesis for these populations to cater to their unique needs and expectations on the reconstructed knee.

The current study has the following limitations: most of the individuals in this review are young adults (< 40 years) with pristine cartilage and bony anatomy which may not be the expected state of structures encountered during TKR surgery. Nevertheless, the current review had representations from 18-year old individuals to elderly subjects in their 80 s. Moreover, morphometric measurements done using MRI may not reflect the actual bony structures. Nonetheless, there is good literature to support the use of CT and MRI for the purpose of morphometry.

## Conclusion

The morphometry of the Filipino knee is comparable to that of other Asian ethnicities but possesses subtle variances. Most of the available TKR prostheses in the Philippine and Asian markets can be fitted to the Filipino knee but the surgeon and the patient must be cautioned about the possible underhang on the mediolateral aspects. The best approach is to come up with a prosthesis aptly suited for the Filipino knees.

## Data Availability

The datasets used and/or analyzed during the current study are available from the corresponding authors on reasonable request.

## Appendix

Tables 12 and 13

**Table 12:** Inter-rater Reliability, *n* = 675

**Parameter**	**Reliability Measure** *using Pearson r Product Moment Correlation*
fAP	0.308^a^ (< 0.001)
fMAP	0.676 ^a^ (< 0.001)
fLAP	0.662 ^a^ (< 0.001)
fML	0.824 ^a^ (< 0.001)
fAML	0.693 ^a^ (< 0.001)
fPML	0.669 ^a^ (< 0.001)
tAP	0.725 ^a^ (< 0.001)
tML	0.333 ^a^ (< 0.001)
tMAP	0.506 ^a^ (< 0.001)
tLAP	0.695 ^a^ (< 0.001)
pH	0.545 ^a^ (< 0.001)
pW	0.832 ^a^ (< 0.001)
pT	0.173 ^a^ (< 0.001)

Values are presented in Correlation Coefficient (*P-*Value); ^a^Significance was set at *P* < *0.05.*

**Table 13:** Correlation of the knee morphometric parameters (*n* = 675)

	**FAP**	**FMAP**	**FLAP**	**FML**	**FAML**	**FPML**	**FAR**	**TAP**	**TML**	**TMAP**	**TLAP**	**TAR**	**PH**	**PW**
FMAP	0.585 ^a^ (< 0.001)													
FLAP	0.659 ^a^ (< 0.001)	0.738 ^a^ (< 0.001)												
FML	0.598 ^a^ (< 0.001)	0.731 ^a^ (< 0.001)	0.741 ^a^ (< 0.001)											
FAML	0.285 ^a^ (< 0.001)	0.465 ^a^ (< 0.001)	0.440 ^a^ (< 0.001)	0.590 ^a^ (< 0.001)										
FPML	0.307 ^a^ (< 0.001)	0.469 ^a^ (< 0.001)	0.423 ^a^ (< 0.001)	0.570 ^a^ (< 0.001)	0.651 ^a^ (< 0.001)									
FAR	0.134 ^a^ (< 0.001)	0.019 (0.627)	0.038 (0.320)	0.636 ^a^ (< 0.001)	0.360 ^a^ (< 0.001)	0.332 ^a^ (< 0.001)								
TAP	0.397 ^a^ (< 0.001)	0.650 ^a^ (< 0.001)	0.577 ^a^ (< 0.001)	0.655 ^a^ (< 0.001)	0.583 ^a^ (< 0.001)	0.615 ^a^ (< 0.001)	0.243 ^a^ (< 0.001)							
TML	0.297 ^a^ (< 0.001)	0.437 ^a^ (< 0.001)	0.434 ^a^ (< 0.001)	0.519 ^a^ (< 0.001)	0.463 ^a^ (< 0.001)	0.488 ^a^ (< 0.001)	0.261 ^a^ (< 0.001)	0.579 ^a^ (< 0.001)						
TMAP	0.434 ^a^ (< 0.001)	0.592 ^a^ (< 0.001)	0.566 ^a^ (< 0.001)	0.639 ^a^ (< 0.001)	0.673 ^a^ (< 0.001)	0.665 ^a^ (< 0.001)	0.265 ^a^ (< 0.001)	0.696 ^a^ (< 0.001)	0.496 ^a^ (< 0.001)					
TLAP	0.452 ^a^ (< 0.001)	0.665 ^a^ (< 0.001)	0.605 ^a^ (< 0.001)	0.687 ^a^ (< 0.001)	0.649 ^a^ (< 0.001)	0.650 ^a^ (< 0.001)	0.261 ^a^ (< 0.001)	0.753 ^a^ (< 0.001)	0.477 ^a^ (< 0.001)	0.774 ^a^ (< 0.001)				
TAR	0.017 (0.663)	0.037 (0.343)	0.071 (0.067)	0.120 ^a^ (0.002)	0.043 (0.261)	0.075 (0.051)	0.129 ^a^ (0.001)	0.123 ^a^ (0.001)	0.784 ^a^ (< 0.001)	-0.099 ^a^ (0.010)	-0.128 ^a^ (0.001)			
PH	0.384 ^a^ (< 0.001)	0.469 ^a^ (< 0.001)	0.445 ^a^ (< 0.001)	0.530 ^a^ (< 0.001)	0.579 ^a^ (< 0.001)	0.605 ^a^ (< 0.001)	0.252 ^a^ (< 0.001)	0.580 ^a^ (< 0.001)	0.397 ^a^ (< 0.001)	0.644 ^a^ (< 0.001)	0.644 ^a^ (< 0.001)	-0.024 (0.528)		
PW	0.405 ^a^ (< 0.001)	0.564 ^a^ (< 0.001)	0.560 ^a^ (< 0.001)	0.656 ^a^ (< 0.001)	0.687 ^a^ (< 0.001)	0.645 ^a^ (< 0.001)	0.313 ^a^ (< 0.001)	0.622 ^a^ (< 0.001)	0.441 ^a^ (< 0.001)	0.651 ^a^ (< 0.001)	0.661 ^a^ (< 0.001)	0.024 (0.535)	0.653 ^a^ (< 0.001)	
PT	0.233 ^a^ (< 0.001)	0.282 ^a^ (< 0.001)	0.222 ^a^ (< 0.001)	0.280 ^a^ (< 0.001)	0.259 ^a^ (< 0.001)	0.269 ^a^ (< 0.001)	0.122 ^a^ (0.002)	0.326 ^a^ (< 0.001)	0.211 ^a^ (< 0.001)	0.330 ^a^ (< 0.001)	0.333 ^a^ (< 0.001)	-0.006 (0.881)	0.371 ^a^ (< 0.001)	0.333 ^a^ (< 0.001)

Values are presented in Correlation Coefficient (*P*-Values); ^a^ Significance was set at *P* < *0.05.*

## Abbreviations

TKR: Total knee replacement
AP: Anteroposterior
ML: Mediolateral
fAP: Femoral anteroposterior distance
fML: Femoral mediolateral distance
fLAP: Femoral lateral anteroposterior distance
fMAP: Femoral medial anteroposterior distance
fAML: Femoral anterior mediolateral distance
fPML: Femoral posterior mediolateral distance
tAP: Tibial anteroposteriordistance
tML: Tibial mediolateral distance
tLAP: Tibial lateral anteroposterior distance
tMAP: Tibial medial anteroposterior distance
